# Editorial: Rising stars in parasite and host 2022

**DOI:** 10.3389/fcimb.2022.1054309

**Published:** 2022-12-19

**Authors:** Mariana De Niz, Daniel A. Gold, Sudhir Kumar, Fred David Mast, Dave Richard, Maria L. Simões

**Affiliations:** ^1^ Department of Parasites and Insect Vectors, Institut Pasteur, Paris, France; ^2^ St. Edward’s University, School of Natural Sciences, Austin, TX, United States; ^3^ Center for Global Infectious Disease Research, Seattle Children’s Research Institute, Seattle, WA, United States; ^4^ Centre de Recherche du Centre Hospitalier Universitaire (CHU) de Québec, Université Laval, Québec, QC, Canada; ^5^ Infectious Disease Research Centre, Université Laval, Québec, QC, Canada; ^6^ Faculty of Infectious and Tropical Diseases, London School of Hygiene and Tropical Medicine, London, United Kingdom

**Keywords:** parasitology, malaria, *Toxoplasma*, *Leishmania*, *Trypanosoma*, *Drosophila*, insect vector

## Abstract

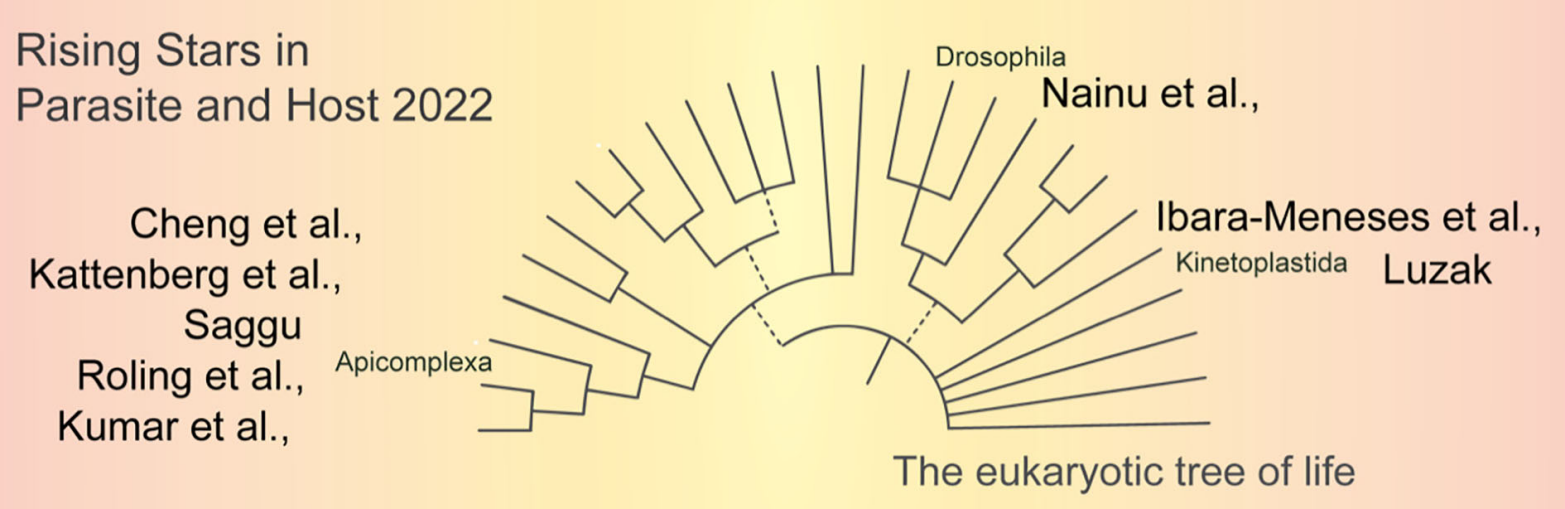

The aim of the collection Rising Stars in Parasite and Host is to highlight the work of the upcoming generation of scientific leaders in parasitology. We received submissions from early career researchers (junior group leaders and postdocs) from around the world, across institutions in Australia, Bangladesh, Belgium, Brazil, Canada, China, Germany, India, Indonesia, Thailand, UK, USA and Vietnam. The topics addressed range from molecular and cellular biology to therapeutics and epidemiological surveillance of Apicomplexan (*Plasmodium* and *Toxoplasma* spp.) and Kinetoplastid (*Trypanosma* and *Leishmania* spp.) parasites, as well as innovative models to study vector-parasite interactions.

## Apicomplexans

*Plasmodium* spp. and *Toxoplasma* spp. are Apicomplexan parasites. *Plasmodium* spp. are transmitted by *Anopheles* mosquitoes, and causative of malaria. Malaria is a major public health concern, causing 241 million cases in 2020, and over 600,000 deaths worldwide (WHO, 2022a). Four separate articles relevant to various aspects of *Plasmodium* biology and tools for epidemiological surveillance were submitted to this collection.

In their Original Research paper, Kumar et al. provide new insights into the profound difference observed in the competitive growth of *Plasmodium falcip*arum parasites, when serum *versus* AlbuMAX is used for their asexual growth in the laboratory. The authors used parasite replicate genetic crosses between *P. falciparum* NF54 clone 3D7 and *P. falciparum* NHP4026 clone, and bulk segregant analysis, to compare genome-wide allele frequency changes in parallel blood stage cultures kept in media containing human serum or media containing AlbuMAX. They found three quantitative trait loci regions associated with the differential parasite growth between the two medium types, in three independent progeny pools. This study shows how the use of parasite crosses combined with bulk segregant analysis will allow systematic dissection of important nutrient acquisition and metabolism pathways, as well as red blood cell invasion pathways, in the most lethal malaria parasite.

Also under the molecular biology umbrella, in their Brief Research Report, Roling et al. focus on the transmission stages of *P. falciparum* and demonstrate the importance of a WD40-repeat protein named PfWLP1 in the exflagellation of male gametocytes. Using a strain where PfWLP1 expression can be conditionally knocked down, the authors show reduced amounts of PfCCp-proteins, components of adhesion complexes localized on the surface of gametocytes. The authors suggest that PfWLP1 forms part of an interaction platform for this adhesion complex and that its absence leads to defects in its assembly and/or integrity. Since PfWLP1 is localized in the parasite cytoplasm, they propose the existence of an unidentified transmembrane linker protein bridging PfWLP1 to the PfCCp complex. Because some of the members of this complex are currently investigated as targets for anti-transmission vaccines, understanding the mechanisms governing their trafficking and assembly is of high interest.

The third submission within the area of molecular and cell biology of *Plasmodium* spp. was an opinion paper by Saggu. In his work, Saggu focuses on the apicoplast, an essential and unique organelle in Apicomplexan parasites which is an attractive target for anti-malaria drugs. He begins by discussing the metabolic relevance of the apicoplast, and the evolutionary history of this organelle – including the loss of the mevalonate dependent pathway for isoprenoid biosynthesis, and the essentiality of various metabolic pathways throughout different stages of the parasites’ life cycle. He discusses the importance of this organelle as an anti-malaria drug target. Interestingly, Saggu frames the discussion on the apicoplast being the result of a continuous degenerative process and raises the hypothesis that if this degenerative process continues, apicoplast replacement in *Plasmodium* parasites will take place, with other compartments taking over this organelle’s only essential function: provision of a functional mevalonate independent pathway.

Moving on from molecular biology was one submission under the umbrella of *Plasmodium* epidemiology. *Plasmodium vivax* malaria and its impact on public health have been largely underestimated, partly due to the biology of this parasite that results in “hidden” infections. In their Original Research Article, Kattenberg et al. present a new highly-multiplexed deep sequencing assay for *P. vivax* that was validated using isolates from travelers and migrants in Belgium, as well as samples collected in Vietnam. The Pv AmpliSeq assay achieved good spatial specificity for between-country of origin prediction using the 33-SNP vivaxGEN-geo panel that targets rare alleles specific for certain geographical regions. The assay also used a newly designed 42-SNP within-country barcode for analysis of parasite dynamics in Vietnam. Pv AmpliSeq performed well for multiple use cases drawing conclusions on parasite diversity, distribution of parasite resistance, and dynamics of parasite populations, and has the potential to be adaptable to application in different geographical regions.

Unlike the vector-borne *Plasmodium* spp.*, Toxoplasma gondii* is transmitted *via* the foodborne route, and is causative of toxoplasmosis. *T. gondii* infection causes a wide range of clinical symptoms and is estimated to affect more than three quarters of the worldwide population (CDC, 2018). At particular risk are pregnant women and immunocompromised individuals (Pittman et al., 2014). One article, focusing on therapeutic strategies against *T. gondii*, was submitted to this collection. In their Review Article, Cheng et al. review the molecular interplay between *T. gondii* and the host innate immune response, particularly focusing on host autophagic- and interferon gamma-mediated responses. The authors carefully delineate how these interactions differ in both a *Toxoplasma* strain- and host cell type-dependent manner. Finally, the authors have bioinformatically analyzed and identified candidate known autophagy-modulating small molecules to serve as possible novel toxoplasmocidal drugs.

## Kinetoplastids

*Leishmania* spp. and *Trypanosoma* spp. are Kinetoplastid parasites. *Leishmania* spp. are transmitted by the bite of infected phlebotomine sandflies. They cause three main forms of leishmaniasis, namely, cutaneous, muco-cutaneous and visceral leishmaniasis. An estimate of 700,000 to 1 million new cases occur every year (WHO, 2022b). One article, focusing on therapeutic strategies against *Leishmania infantum* (causative of visceral leishmaniasis), was submitted to this collection. In their Original Research Paper, Ibarra-Meneses et al. present a novel protocol based on thermal proteomic profiling (TTP) (Mateus et al., 2020), and identify novel direct and off-target players involved in resistance and susceptibility to current anti-leishmanial drugs (namely, antimony, miltefosine, and amphotericin B). TTP is based on the physical principle that proteins become more resistant to heat-induced denaturation when coupled to a ligand (such as drugs, nucleic acids, and other proteins), and this thermal change is quantifiable by mass-spectrometry. Thus, the authors present the first ‘meltome’ of *L. infantum*, and propose this as a promising addition to the anti-leishmanial chemotherapy toolkit.

*Trypanosoma* spp. are transmitted by the bite of infected tse-tse flies (*Glossina* spp.). They are able to infect a plethora of animals, and cause Human African Trypanosomiasis (HAT), or sleeping sickness in humans, and nagana in mammals. Around 600 cases were reported in humans in 2020 (WHO, 2022c) One article, focusing on targets against *T. brucei* immune evasion, was submitted to this collection. In their Opinion article, Luzak begins by highlighting the fact that pathogens from diverse phyla use antigenic variation as a strategy for survival. They discuss the importance of identifying common principles of antigenic variation as a strategy to combat infectious diseases, and focus on nuclear condensates as potential targets to manipulate parasite immune evasion. Luzak discusses that nuclear condensates (Bhat et al., 2021) (i.e. functional compartments within the nucleoplasm that lack a lipid membrane and can therefore create specific microenvironments on demand) have a regulatory potential that remains to be explored in biology. They summarize main discoveries in *Trypanosoma* spp. research, from the description of the first nuclear condensate, to multi-condensate assembly, and then raise important questions, including the relevance of temperature on antigen expression and switching. Temperature within the vector and mammalian host differs significantly, as it does in infected mammals with fever. Finally, Luzak proposes a combination of molecular and imaging methods which could be used to further explore this avenue in the future.

## Novel models

Vector and rodent models of parasitic infections have been invaluable for research over the last century. One article, evaluating the value of *Drosophila melanogaster* as a model to study vector-borne parasitic diseases, was submitted to this collection. In this Opinion article, Nainu et al. remind us of the utility and feasibility of using the fruit fly *D. melanogaster* as a model for studying medically important vector-borne diseases. This model has been extensively used for the study of bacteria and viruses that cause human diseases, but relatively underexplored in the study of human parasitic diseases. Hence, the authors propose the use of the fruit fly in studies concerning the biology, immunology and pathogenic characteristics of human parasites vectored by insects. About 70% of the genes for human diseases have homology in *Drosophila* which, together with its simpler genetics, makes this model suitable for the application of diverse genetic tools to explore important medical parasitology questions.

## Concluding remarks

Together, the collection of articles submitted to the Research Topic Rising Stars in Parasite and Host highlights the broad range of interests of emerging scientific leaders within the umbrella of parasitology. This includes state-of-the-art methods for diagnosis and drug susceptibility/resistance, epidemiological surveillance, emerging topics in molecular biology for mechanisms including antigenic variation, and the use of insect models for the study of parasite-vector interactions. Without a doubt, this Rising Stars in Parasite and Host collection presents various novel tools to the scientific community, thus highlighting the important link of technology development to address novel scientific questions. This has always been key for the advancement of science, and will clearly play a pivotal role in the future of parasitology.
